# Women’s and men’s perceptions of (not) wearing a bra in public

**DOI:** 10.3389/fpsyg.2026.1797201

**Published:** 2026-04-13

**Authors:** Pavol Prokop, Ivana Tomanová Čergeťová, Jozef Balcerčík

**Affiliations:** 1 Department of Environmental Ecology and Landscape Management, Faculty of Natural Sciences, Comenius University, Bratislava, Slovakia; 2 Institute of Zoology, Slovak Academy of Sciences, Bratislava, Slovakia; 3 MMT Consulting, s.r.o., Bratislava, Slovakia

**Keywords:** breast attractiveness, female infidelity, female self-promotion, mate choice, sexual harassment

## Abstract

**Introduction:**

Human females often use clothing as a strategy for self-promotion to enhance their physical attractiveness to potential mates. The conscious concealment or revelation of intimate body parts can be used to solicit attention; however, little empirical research exists on this topic.

**Methods:**

We investigated the factors influencing the decision to wear a bra using self-reports from Slovak women (*N* = 409) in public and private settings. Furthermore, using visual stimuli, we examined how male (*N* = 277) and female (*N* = 158) raters perceived the attractiveness and faithfulness of women with and without a bra.

**Results:**

We found that being braless was significantly less frequent in public than in private settings. The decision was negatively associated with the fear of sexual harassment and positively associated with smaller, firmer breasts, but not with unrestricted sociosexuality. The braless state was consistently perceived by both men and women as more attractive, yet also as less faithful. Males with a stronger preference for large breasts found the braless state more attractive than those with a lower preference. Finally, male intentions to sexually harass females, as well as an unrestricted sociosexuality in male raters, were positively correlated with the perception of the braless state as a cue of infidelity.

**Discussion:**

Our results suggest that bra-wearing represents a strategic trade-off in self-promotion: while it may dampen sexual attractiveness, it simultaneously protects the wearer from being perceived as infidel.

## Introduction

Human males show a preference for permanent female breasts of average to slightly above-average size and high firmness (Dixson et al., 2011, 2015; Kościński, 2019; Groyecka et al., 2017; Prokop et al., 2020; Pazhoohi et al., 2020). Male preferences and the sexual arousal they experience upon seeing women’s breasts are innate rather than culturally determined (Stefańczyk et al., 2025). Breast firmness has been proposed as a reliable indicator of youth and nulliparity, serving as a direct cue to a woman’s fertility (Marlowe, 1998; Pazhoohi et al., 2020). This perspective suggests that permanent breasts evolved to make this age-related signal more discernible (Havlíček et al., 2017; Kościński, 2019). An alternative hypothesis proposes that after the adoption of bipedalism, protruding breasts gained salience by visually mimicking the buttocks from a frontal view, exploiting an existing male perceptual bias (Morris, 1967; Guthrie, 1976). These perspectives are not mutually exclusive but instead suggest that multiple selective pressures may have shaped male preferences.

Male preference for average or above-average size may have been adaptive as it could have signaled sufficient fat reserves. According to the Handicap Principle, this would serve as an honest indicator of a woman’s health and metabolic resources (Zahavi and Zahavi, 1999). Others have suggested this signal was specifically linked to the fat reserves necessary for successful lactation and child-rearing (Cant, 1981; Gallup, 1982). Importantly, these preferences are context-dependent, with stronger preferences for large breasts observed among men pursuing short-term mating strategies (Zelazniewicz and Pawlowski, 2011).

Human males show a preference for permanent female breasts of average to slightly above-average size and high firmness (Dixson et al., 2011, 2015; Kościński, 2019; Groyecka et al., 2017; Prokop et al., 2020; Pazhoohi et al., 2020). Breast firmness is a reliable indicator of youth and nulliparity, serving as a direct cue to a woman’s fertility (Marlowe, 1998; Pazhoohi et al., 2020). This perspective suggests that permanent breasts evolved to make this age-related signal more discernible (Havlíček et al., 2017; Kościński, 2019). An alternative hypothesis proposes that after the adoption of bipedalism, protruding breasts gained salience by visually mimicking the buttocks from a frontal view, exploiting the existing male perceptual bias (Morris, 1967; Guthrie, 1976). Male preference for average or above-average size may have been adaptive, as it could have signaled sufficient fat reserves. According to the Handicap Principle, this would serve as an honest indicator of a woman’s health and metabolic resources (Zahavi and Zahavi, 1999). Others have suggested that this signal is specifically linked to the fat reserves necessary for successful lactation and child-rearing (Cant, 1981; Gallup, 1982). This preference appears to be most pronounced for large breasts, particularly among men pursuing a short-term mating strategy (Zelazniewicz and Pawlowski, 2011).

From an evolutionary perspective, clothing functions as a self-promotion strategy designed to enhance an individual’s mate value and increase sexual success (Davis and Arnocky, 2022). This type of appearance enhancement reflects a competitive effort to become more attractive to potential partners by aligning with preferred physical standards, ultimately increasing the chances of successful mating (Buss and Schmitt, 2019; Gangestad and Scheyd, 2005). However, clothing serves a dual role; while it can accentuate, it can also function to conceal. The degree of concealment of intimate body parts can act as a cue to sexual restrictiveness and fidelity (Pazhoohi and Kingstone, 2020; Pazhoohi, 2024), whereas provocative clothing is typically associated with sexual availability (Grammer et al., 2004; Wolfendale, 2016). However, adorning such sexualized garb can create perceptions of women as promiscuous and lacking moral agency, which in turn leads to sexual objectification and a greater risk of sexual aggression (Blake et al., 2016). For instance, men project more sexual and positive emotions onto women when they display erect nipples and perceive them as more likely to engage in sexual behavior (Burch and Widman, 2021; Widman and Burch, 2024). Therefore, the choice of whether to wear a bra presents a unique context for examining this strategic trade-off in self-promotion. On the one hand, not wearing a bra can increase sexual attractiveness by making nipple erection more visible, a cue for immediate sexual interest. Conversely, wearing a bra can improve the visual appearance of the breasts, making them look firmer and more youthful (Greggianin et al., 2018; Zhong et al., 2023), potentially creating a supernormal stimulus for fertility cues. Such stimuli are exaggerated versions of natural traits, which can elicit a stronger response from the receiver than the original cue for which the preference evolved (Gray and McKinnon, 2007).

Clothing choices, however, do not only influence attractiveness, they also shape social perceptions and judgments. Provocative dressing can lead to harmful stereotypes and victim-blaming attitudes, suggesting that women bear responsibility for the sexual aggression they face (Johnson and Workman, 1992; Wolfendale, 2016). This objectification extends to the most intimate aspects of women’s attire, where, in our opinion, the deliberate choice to forgo a bra can be interpreted as an explicit signal of sexual availability and vulnerability. This perspective is supported by research linking hostile sexism—a dimension of sexism characterized by antagonistic beliefs about women—to specific preferences for female attractiveness (Lennon et al., 2017). These interpretations are not uniform but depend on inter-individual differences. Men with higher levels of hostile sexism demonstrate a stronger preference for larger breast size (Pazhoohi et al., 2020; Swami and Tovée, 2013). This preference is consistent with traditional and objectifying views of women’s bodies (Forbes et al., 2007; Swami et al., 2010a,b).

Building on this, we propose that the same hostile sexist attitudes that favor larger breasts also shape the interpretation of their presentation. Consequently, women whose appearance, such as a visibly braless state, defies normative expectations or is perceived as provocative, are often viewed as sexual objects. This objectification can lead to increased vulnerability to harassment (Blake et al., 2016). Thus, the same cues that enhance attractiveness may simultaneously increase perceived sexual availability and associated social risks.

The trade-off in self-promotion suggests that women strategically manage their sexual signals to enhance male attention (Buss, 2017; Buss and Schmitt, 1993). For instance, when anticipating an interaction with an attractive potential opposite-sex partner, women showed preferences for wearing the color red and high-heel shoes, that is, sexual signals that increase the perceived attractiveness of the bearer (Prokop and Švancárová, 2020; Elliot et al., 2013).

Individual differences in male psychology may moderate these perceptions. Specifically, men with an unrestricted sociosexual orientation (SOI) consistently demonstrate higher levels of rape myth acceptance, adversarial sexual beliefs, and a history of sexual coercion (Yost and Zurbriggen, 2006; Kennair and Bendixen, 2012). This suggests that unrestricted men are more likely to initiate unwanted sexual advances and interpret ambiguous social signals as sexual invitations (Haselton, 2003). Given that pornography consumption correlates with an unrestricted SOI (Kennair and Bendixen, 2012; Prokop, 2015), it may also contribute to the development of a preference for cues such as a braless state, which are often sexualized in such media. Moreover, sexually unrestricted men assign greater importance to women’s physical attractiveness compared to sexually restricted men (Sacco et al., 2009; Simpson and Gangestad, 1992; Swami et al., 2009). Consequently, we predict that men with a high SOI will be particularly sensitive to the signal of a braless state. Men are expected to rate such women as significantly more sexually attractive. Crucially, they also see them as more likely to cheat and be sexually exploitable—a view that encourages harassment by portraying targets as available and less resistant (Craib et al., 2024; Kennair and Bendixen, 2012).

### The present study

The present study is based in an evolutionary framework in which physical traits and appearance-enhancing behaviors function to communicate information about reproductive value, mating strategy, and sexual intent. Within this framework, the decision to wear or forgo a bra represents a strategic trade-off between enhancing morphological cues associated with fertility (e.g., firmness) and increasing the salience of dynamic sexual signals (e.g., nipple visibility). It should be pointed that clothing behavior and its perception are also influenced by proximate social and cultural factors, such as fashion norms, comfort, and body image. The current study focuses specifically on the signaling functions of these behaviors, while acknowledging that multiple levels of explanation may contribute to the observed patterns.

Accordingly, this study integrates two complementary levels of analysis:

(1) Sexual signaling (women’s behavior): We examine whether women’s bra-wearing frequency is predicted by their underlying morphological traits and mating orientation. Specifically, we hypothesize that:

*H1:* Women displaying morphological cues associated with higher fertility and reproductive value (i.e., larger and firmer breasts) will report lower bra-wearing frequency.

*H2:* Women with a more unrestricted sociosexual orientation (SOI) will report lower bra-wearing frequency, reflecting a strategy to enhance sexual signaling and attractiveness.

(2) Signal perception (mens’ judgments): We investigate how the absence of a bra influences male perceptions of women’s attractiveness, sexual openness, and fidelity. Based on evolutionary and social-cognitive perspectives, we hypothesize that:

*H3:* Women depicted without a bra will be perceived as more sexually attractive compared to women wearing a bra.

*H4:* Women depicted without a bra will be perceived as more sexually open and more likely to engage in infidelity.

(3) Moderating role of individual differences in men: We further examine whether these perceptions are moderated by male individual differences, particularly sociosexual orientation and hostile sexism. We hypothesize that:

*H5:* Men with a more unrestricted SOI will show stronger increases in perceived attractiveness, sexual openness, and infidelity in response to a braless presentation.

*H6:* Men higher in hostile sexism will be more likely to interpret a braless state as a cue of sexual availability and vulnerability.

(4) Consequences for perceived risk and social outcomes: Finally, we examine whether these perceptions translate into expectations about social consequences:

*H7:* A braless presentation will be associated with increased anticipated vulnerability to harassment, particularly among men scoring high in hostile sexism.

In addition to main predictors, age and education were entered as standard demographic controls. Pornography consumption was included as an additional control because it influences sexual behavior (Rogala and Tydén, 2003). Women’s self-esteem is linked to breast satisfaction (Swami et al., 2015); thus, we predict it positively correlates with a braless state. Media exposure is linked to breast dissatisfaction (Swami et al., 2020); we expect greater exposure to predict lower likelihood of not wearing a bra in public. Finally, silicone implants were included because they increase breast size and satisfaction with breast appearance (Chao et al., 2016).

## Methods

### Participants

The participants were *N* = 686 (409 females) heterosexual Slovak adults between the ages of 18 and 61 years (M = 34.64 years, SE = 0.32). Additional non-heterosexual men (*N* = 8) and women (*N* = 22) were not included in the analyses. Men were slightly older on average (35.58 years) than women (34.00 years), but both groups had similar standard deviations of approximately 8.3 years. Participants were primarily recruited through advertisements placed on social networking sites, specifically Facebook, Linkedin, and Instagram, and via emails distributed to university students targeting both the student and general public. The advertisements and information provided to potential participants described the study as investigating attitudes toward human sexual behavior. Participation was entirely voluntary, and no financial or other incentives were provided. Following the initial recruitment, participants were asked to recruit additional volunteers from their acquaintances using the snowball sampling method (Goodman, 1961). The participants were not aware of the research-specific hypotheses.

### Research instruments

#### Sociosexuality

To assess attitudes toward sexual behavior, the Revised Sociosexual Orientation Inventory (SOI-R; Penke and Asendorpf, 2008; Cronbach *α* = 0.81) was used. This is a 9-item scale which provides an overall measurement of sociosexual orientation (example statement: “With how many different partners have you had sex within the past 12 months?”) as well as three subscales: the attitude subscale measures the participant’s disposition toward short-term sexual encounters; the behavior subscale measures the number of casual sex partners and the frequency of change in partners; and the desire subscale measures the frequency of sexual fantasies or arousal in relation to potential mates with whom the individual is not currently in a committed relationship with. A high SOI-R score indicates an unrestricted sociosexual orientation, in other words, a propensity to engage in more short-term sexual relationships. SOI was administered to both sexes.

### Pornography consumption

Consumption of pornography was measured with a single item modified after Hald (2006). “How frequently do you watch pornographic films?” Possible responses were 1 = never, 2 = 1–2 times per month, 3 = at least once per week, 4 = 4–5 times per week, 5 = daily. Pornography consumption was asked to both sexes.

### Sexual harassment

Fear of sexual harassment in women (Cronbach’s α = 0.94) and intentions to engage in sexual harassment in men (Cronbach’s α = 0.92) were assessed using 14 items slightly modified from Guarderas et al. (2023), examining verbal, nonverbal, and physical sexual harassment. Women were asked to indicate the likelihood of being afraid of 14 harassing situations in a hypothetical scenario when they would go to work without a bra. Each situation was scored from 1 (absolutely not afraid) to 5 (absolutely afraid). Example of item is:

How much would you be afraid from receiving messages, calls, mails, notes, sms, chats with unwanted sexual content? Men received the same items, modified to assess their own behavior in case of having been in contact with a women not wearing a bra in work: How much is it likely that you will send messages, calls, mails, notes, sms, chats with unwanted sexual content? (1 = absolutely unlikely, 5 = absolutely likely).

### Visual stimuli of breast size and shape

The breast size stimuli were adopted from Swami et al. (2015). The instrument consists of 14 computer-generated images of women with increasing breast sizes. Images were presented in grey scale and without facial features to minimize the impact of these features on ratings. Female participants were asked to rate the image that most closely matched their current breast size and the image they would most like to possess, with responses made on a 14-point scale (1 representing the figure with the smallest breast size and 14 representing the figure with the largest breast size) to investigate the actual breast size. The participants were also asked to choose one picture that they desired as an ideal breast size. An index of breast size satisfaction (hereafter “breast dissatisfaction pictures”) was computed as the absolute difference between ideal and current breast size ratings. Higher scores reflect greater breast size dissatisfaction, regardless of the direction of dissatisfaction.

The stimuli on breast shape variation were redrawn from Rawson and Brooks (1984) and consisted of four profile drawings depicting gradually decreasing age-related firmness (high, moderate, rather low, and low). Participants were asked to choose one of the drawings that most resembled their own breast. The scoring of the drawings was reversed (high firmness = 4, low firmness = 1).

Men were also shown breast size images (Swami et al., 2015) and asked which size they considered most sexually attractive (single choice).

### Appearance and weight satisfaction

Consistent with Frederick et al. (2016), women assessed their satisfaction with their physical appearance and weight using two distinct items (1 = Extremely dissatisfied, 7 = Extremely satisfied). Although single-item measures may not capture the full complexity of body image, previous studies have shown that these measures demonstrate adequate convergent validity (Sandhu and Frederick, 2015). Higher scores were associated with greater breast satisfaction (hereafter referred to as “breast satisfaction items”).

### Media exposure

To measure exposure to Western and local mass media, we adapted the media exposure scale developed by Swami et al. (2020). The scale consists of eight items that asked about the frequency of exposure to Western (four items) and local (four items) television shows, movies, magazines, and Internet sites. All items were rated on a 5-point scale (1 = less than once a month, 2 = once or twice a month, 3 = once a week, 4 = several times a week, 5 = every day) (Cronbach’s *α* = 0.65). Higher scores were associated with greater exposure to the media. Media exposure items were administered to both sexes in this study.

### Self-esteem

We adopted the Single-Item Self-Esteem Scale (SISE; Robins et al., 2001), in which participants are asked to rate the statement “I have high self-esteem” on a 7-point scale (1 = Not very true of me, 7 = Very true of me). The SISE scores have been shown to have adequate construct validity (Robins et al., 2001). SISE was administered to both sexes of participants.

### Silicon implants

Women were asked single dichotomous question “Do you have silicon implants?” (yes/no).

### Self-reported wearing a bra

Women were asked two questions: How frequently do you wear a bra in public/at home during the summer? We chose public and home environments because we expected that fear of sexual harassment would influence bra-wearing predominantly in public rather than at home. Possible responses were 1 = 25x per months and more, 2 = 11–24 times per month, 3 = 6–10 times per month, 2–5 times per months, 1 = never. We were mainly interested in not wearing a bra; thus, higher scores indicated less frequent wearing of a bra.

### Visual preferences for (not) wearing a bra

The participants were asked to provide two self-photographed images of their torso while wearing a white T-shirt—one image with a bra and one without a bra from the same distance. The women were also asked to provide their breast size. We received 17 submissions and selected 10 pairs of images for use in the study. The selection criteria were based on picture quality (e.g., pictures taken from the lateral side instead of the front side were not included) and breast size diversity. Regarding the latter, we attempted to capture the highest breast diversity possible; thus, we selected images with apparently dissimilar breast sizes. The breast sizes involved in the study were A1, A3, A4, B2, B3, C1, C2, D1, D2, and F1. Participants were presented with 10 pairs of these images and, using a forced-choice paradigm, were asked to select the image in each pair in which they perceived themselves as more sexually attractive. The same pairs were then shown again in a randomized order, and the participants were asked to choose the image in which they perceived greater fidelity in the depicted woman.

This task was administered to all male participants and a subsample of women (*N* = 158) who completed all other study tasks. Initially, we were concerned that including these image-based tasks would lengthen the questionnaire and potentially reduce the participation rates among women. However, subsequent feedback indicated that participants were willing to complete the questionnaire with the image pairs included in the study. Consequently, we incorporated this component into the final study design.

To validate the images, we individually interviewed an additional sample of university students (*N* = 10; 6 were men) of comparable age (M = 26.8 years, SE = 1.35, range = 21–36) to examine whether raters correctly discriminated between images of women with and without a bra using a closed-response question. Pairs of pictures showing women wearing a bra and not wearing a bra were presented on a 22-inch monitor, and the order of the pictures was randomized. No time limit was imposed for evaluating the pictures. Each student was asked which picture in each pair depicted the braless state (left or right), and the responses were recorded by the interviewer.

### Procedure

The study was conducted online, providing participants with a high level of privacy and anonymity (Robertson et al., 2018). This feature is especially beneficial for the present research, as many of the questions involve highly personal topics such as sexual abuse. The participants were initially asked demographic questions, namely age, gender, sexual orientation (heterosexual, homosexual, lesbian, bisexual, other), and highest level of education received (1 = basic school, 2 = high school, 3 = bachelor’s degree, 4 = master’s degree, 5 = PhD or higher). They then responded to visual tasks, followed by questionnaires. The images on each slide were randomly ordered.

### Statistical analyses

Generalized Linear Mixed Models (GLMMs) were used for statistical analyses. For women, the reported frequency of (not) wearing a bra in public and at home was defined as an ordinal dependent variable in two separate GLMMs, with participant identity (ID) defined as a random effect. Independent predictors included Age, Education Level, Self-esteem score, SOI score, Media Exposure score, Sexual Harassment score, Breast Size preference, Relationship Status, reported pornography consumption frequency, reported breast shape, silicone implants (or not), and breast satisfaction scores from pictures and items.

For men, a GLMM with a binomial dependent variable was used to examine the preferences for pictures of women with and without a bra. The participant ID and picture ID were defined as random effects. The predictors included reported preferences for breast size, participant age, education level, relationship status, sexual harassment, media exposure, pornography consumption, SOI score, and self-esteem. Sex differences were examined using Mann–Whitney U-tests.

To avoid multicollinearity in our statistical models, we centered all continuous predictor variables by subtracting the mean from each value, resulting in variables with a mean of zero while preserving the original scale and variance (Aiken and West, 1991). This centering procedure reduces non-essential multicollinearity between predictors and interaction terms, improves the interpretability of the main effects in the presence of interactions, and enhances numerical stability without altering the underlying relationships in the data (Cohen et al., 2003). The Variance Inflation Factor (VIF) was used to examine the degree of multicollinearity among the independent variables. VIF values were below 2.5, suggesting no multicollinearity concerns (Hair et al., 2010). All statistical tests were performed in Python (Seabold and Perktold, 2010).

## Results

### Predictors of (not) wearing a bra in public in women

**Figure 1:**
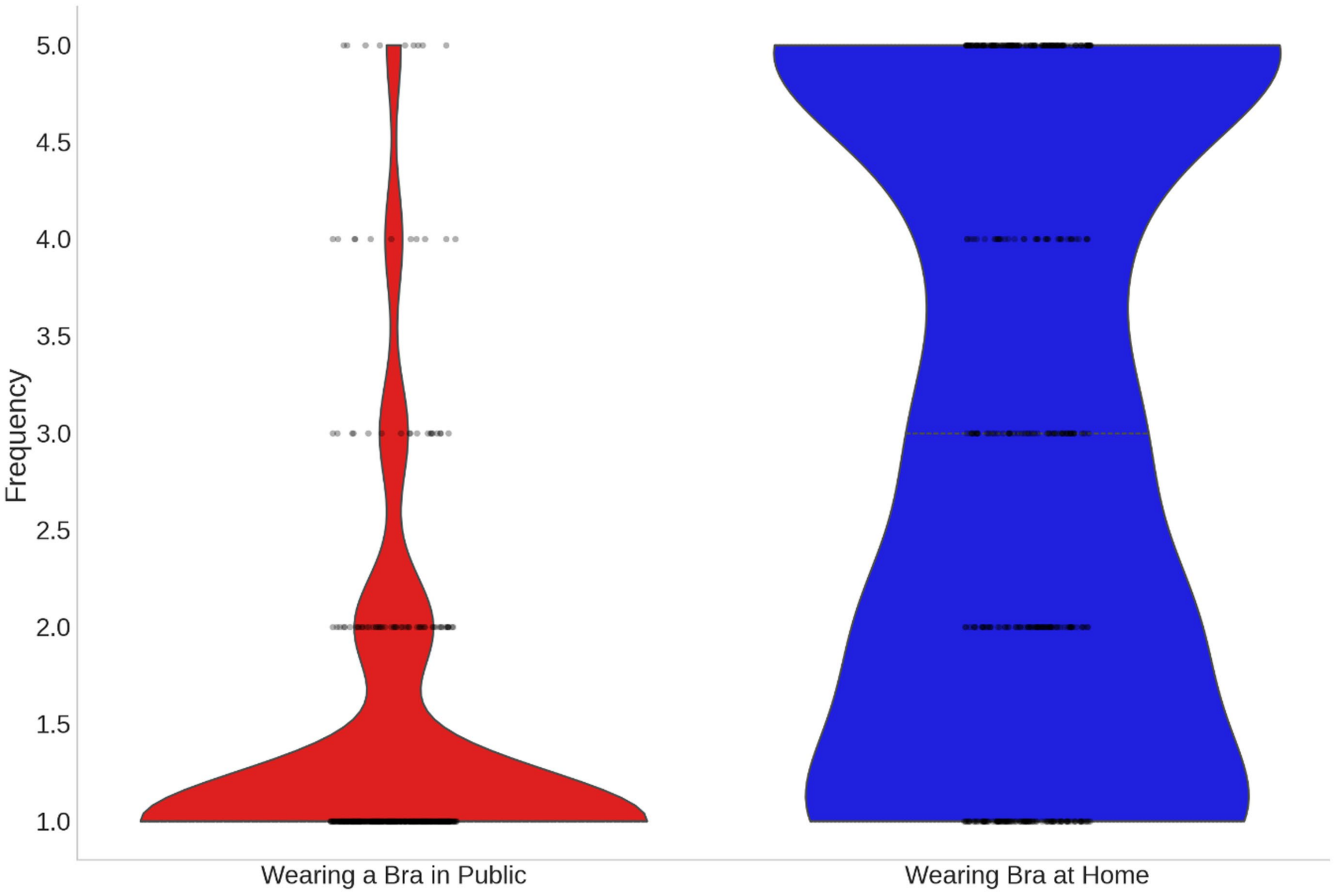
Frequency of wearing a bra in public and at home.

Not wearing a bra in public was infrequent (mean = 1.38, 95% CI [1.29, 1.46], *N* = 409) and differed significantly from a random distribution (one-sample t-test on participant means, t(408) = 37.84, *p* < 0.001). Only 9 women (2.2%) reported almost never wearing a bra, whereas the majority (*N* = 321, 78.5%) reported wearing a bra in public almost every time (Figure 1; Supplementary Table 1).

**Figure 2:**
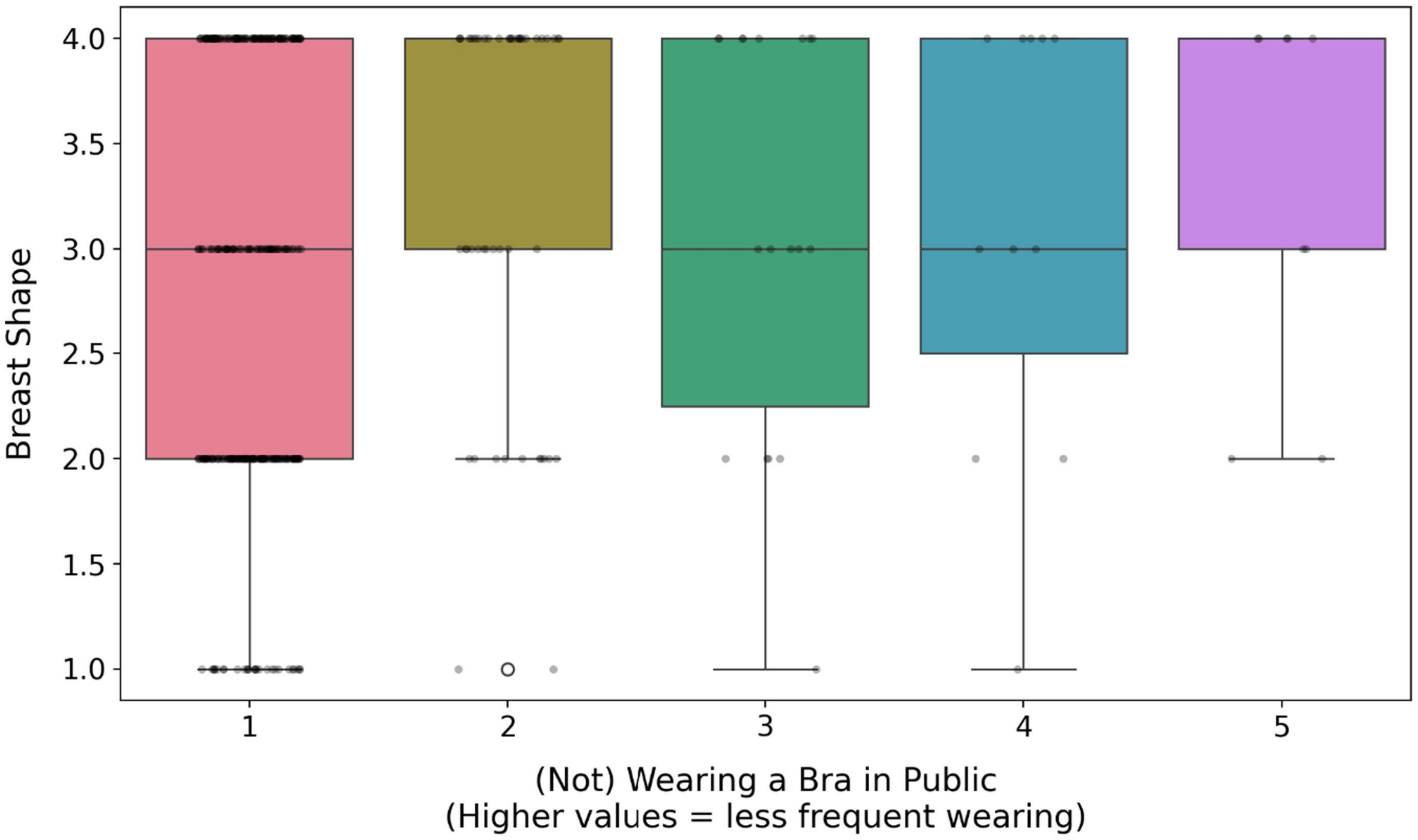
The effect of breast shape on (not) wearing a bra in public. The box shows the interquartile range (IQR), the horizontal line is the median, whiskers extend to 1.5 × IQR beyond the 1st and 3rd quartiles, and points beyond whiskers represent outliers.

**Figure 3:**
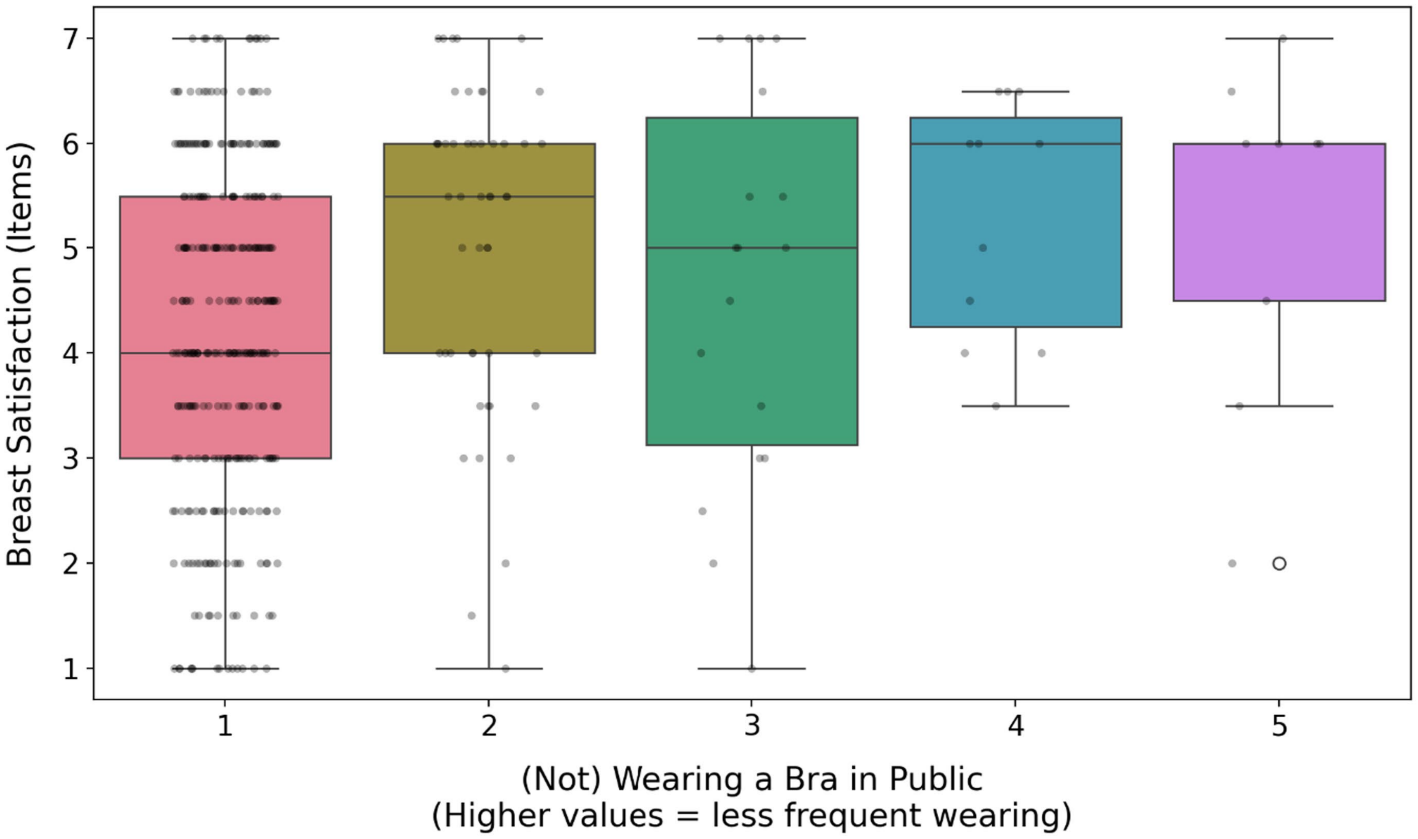
The effect of breast satisfaction (items) on (not) wearing a bra in public. For designations, see Figure 2.

**Figure 4:**
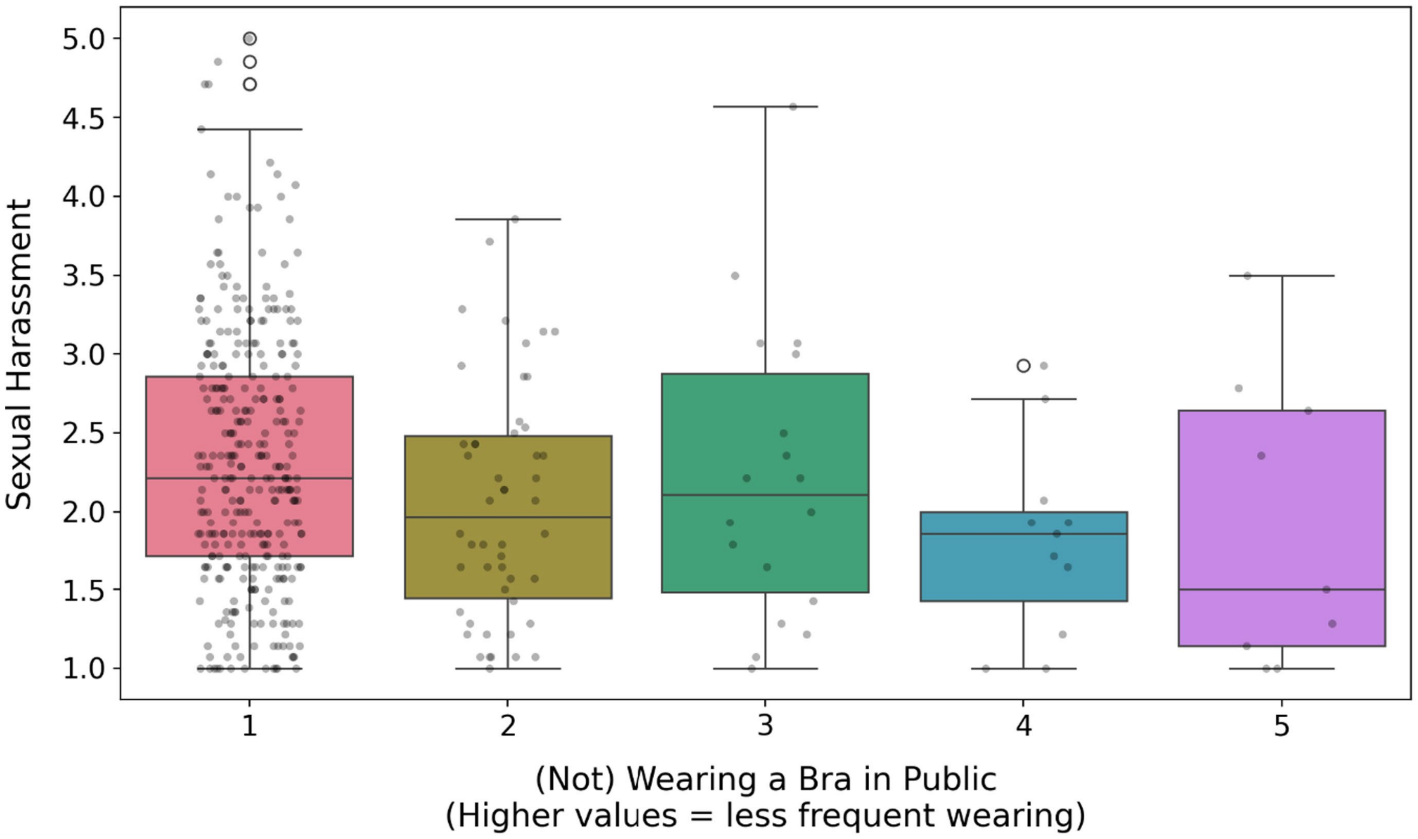
The effect of fear of sexual harassment on (not) wearing a bra in public. For designations, see Figure 2.

**Figure 5:**
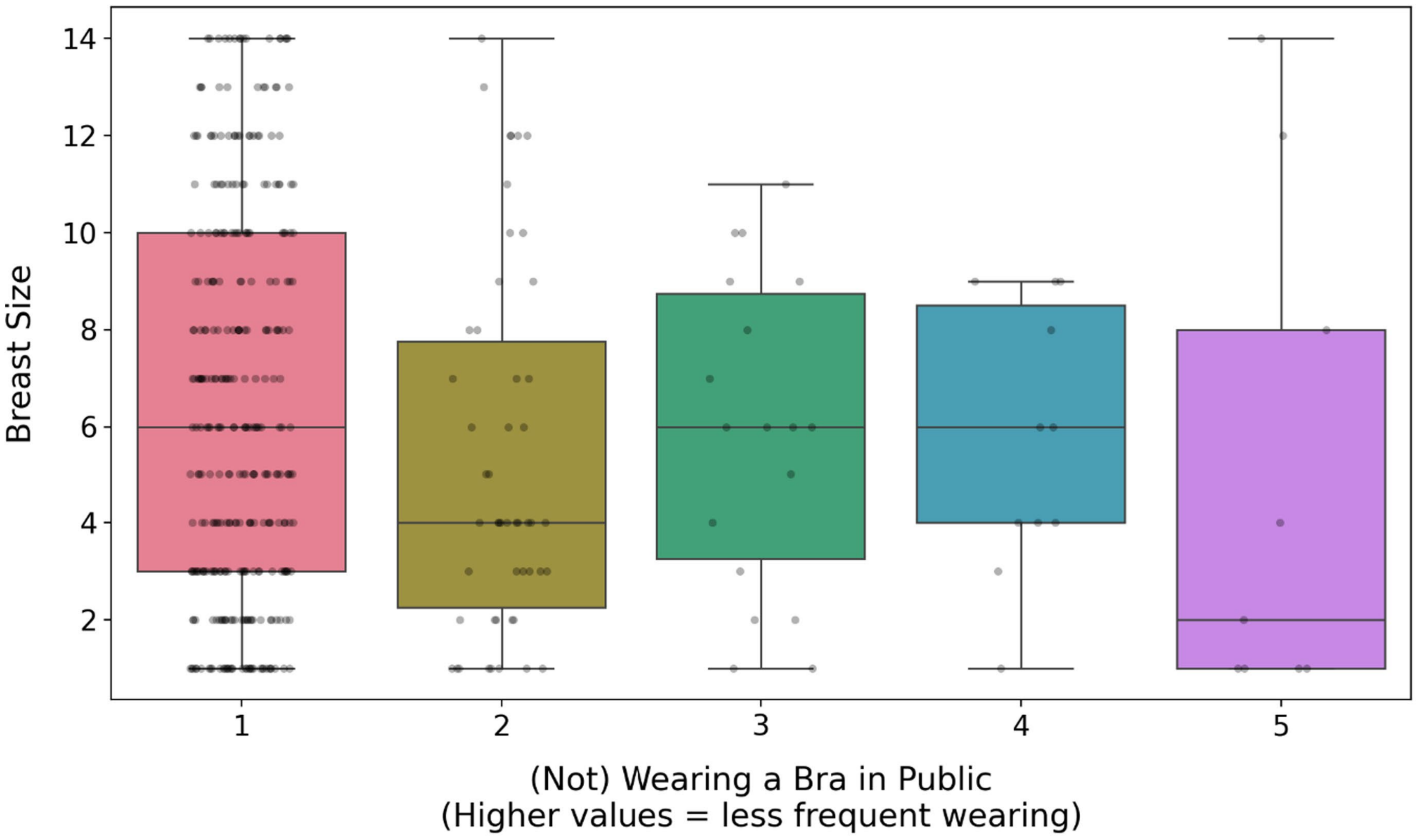
The effect of breast size on (not) wearing a bra in public. For designations, see Figure 2.

Not wearing a bra was significantly and positively predicted by self-esteem, breast shape (firmer breasts; Figure 2), breast satisfaction (item scale; Figure 3; Supplementary Table 2) and the presence of silicone implants. In contrast, it was significantly negatively predicted by age, education level, perceived sexual harassment (Figure 4), media exposure, breast dissatisfaction (photo-based scale) and breast size (Figure 5). Relationship status, SOI and pornography consumption did not influence bra-wearing behavior in public.

Because breast satisfaction (items) and breast dissatisfaction (pictures) were partially correlated (r = 0.16, Supplementary Table 3), we removed each variable in turn and re-ran the GLMM. The direction and significance of both predictors remained stable, indicating that they capture different aspects of breast satisfaction independent of each other.

Removing pornography consumption—which correlated with SOI (r = 0.28, *p* < 0.001)—and then removing SOI did not change the model. A similar procedure was applied to breast size and breast shape (r = −0.61, *p* < 0.001), and again, the direction of effects remained unchanged. The only difference was that the coefficient for breast size became more negative at −0.023 (*p* < 0.001), showing increased statistical significance.

### Predictors of (not) wearing a bra at home in women

Not wearing a bra at home was very frequent (mean = 3.03, 95% CI [2.87, 3.19], N = 409) and did not differ significantly from a random distribution (one-sample *t*-test on participant means, t(408) = 0.33, *p* = 0.742). Approximately equal proportions of participants reported wearing a bra almost never or almost daily at home (Figure 1; Supplementary Table 1).

The strongest positive predictors of not wearing a bra at home were having silicone implants and higher breast satisfaction (items). Larger breast size, fear of sexual harassment, and older age were associated with not wearing a bra at home less frequently (Supplementary Table 4). More educated women reported not wearing bra at home more frequently than less educated women. Women with higher SOI scores (more unrestricted sociosexuality) also reported not wearing a bra at home. Media exposure and breast dissatisfaction (pictures) were negatively associated with wearing a bra at home.

Removing pornography consumption and breast satisfaction measures (items or pictures) did not significantly affected the interpretation of the results.

### Preferences of (not) wearing a bra in men

**Figure 6:**
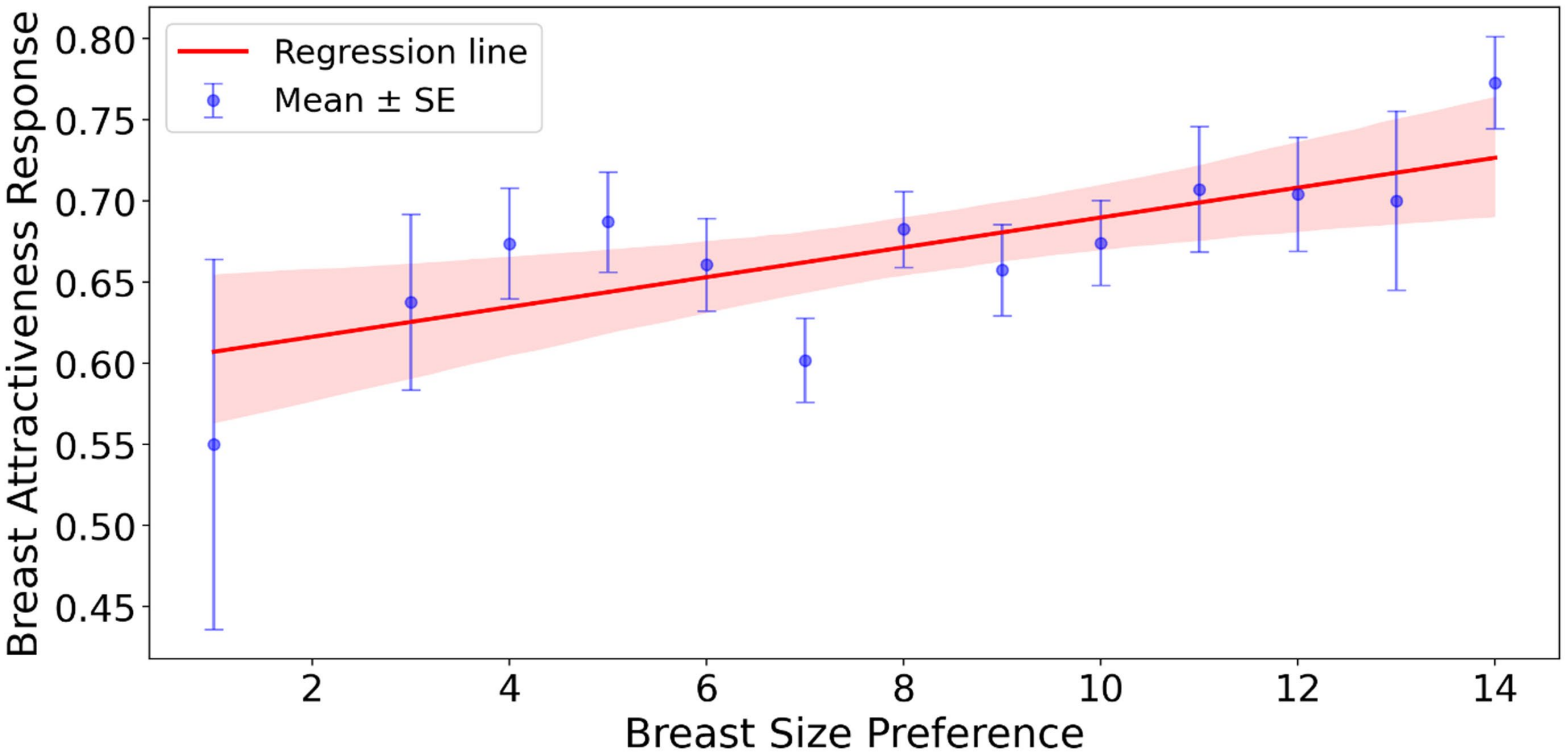
The effect of men’s breast size preference on ratings of attractiveness of women pictures (not) wearing a bra. The shaded area around the regression line in the figure represents the 95% confidence interval for the regression line.

All 10 raters (100%) correctly identified the braless state in the pictures. The mean preference score for seeing women not wearing a bra was 0.67 [95% CI, 0.6564, 0.6914], *N* = 277, significantly above chance (one-sample *t*-test on mean scores: t = 17.46, *p* < 0.001). Thus, men rated breasts without a bra as more attractive than breasts with a bra. Men’s preference for larger breast size was the only predictor of preferring women not wearing a bra (Figure 6). Removing SOI or pornography consumption (which were correlated, did not significantly change this result).

**Figure 7:**
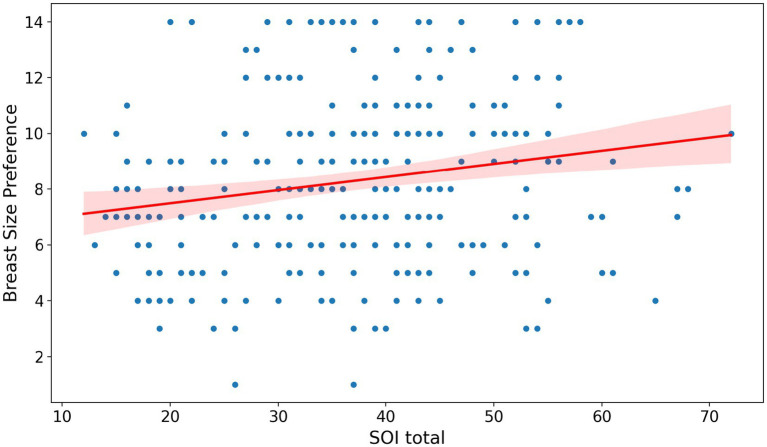
Correlation between men’s sociosexuality (SOI) and breast size preference. The shaded area around the regression line in the figure represents the 95% confidence interval for the regression line.

**Figure 8:**
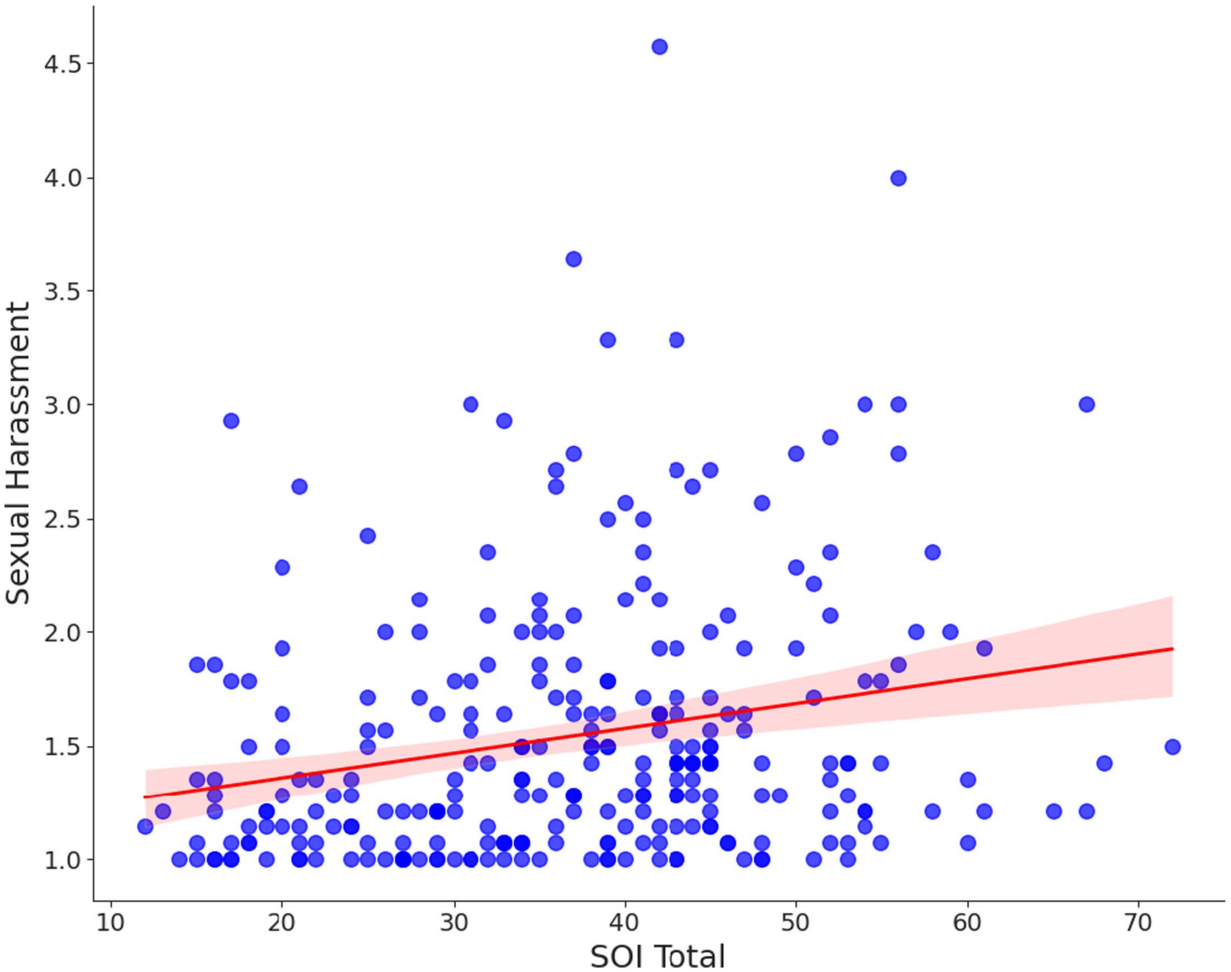
Correlation between men’s sociosexuality (SOI) and intended sexual harassment score. The shaded area around the regression line in the figure represents the 95% confidence interval for the regression line.

Men with more unrestricted sociosexuality preferred larger breasts more strongly (*r* = 0.1933, *p* = 0.0012, Figure 7). Additionally, less sexually restricted men reported higher sexual harassment intention scores (*r* = 0.227, *p* < 0.001, Figure 8). A correlation matrix for all male variables is shown in Supplementary Table Z.

### (Not) wearing bra as a cue of fidelity (men’s perceptions)

Perceived fidelity scores for breasts without a bra were low (mean = 0.15 [95% CI, 0.1328, 0.1591], *N* = 277) but still significantly above chance (one-sample *t*-test on mean scores: *t* = 21.10, *p* < 0.001). Thus, men strongly associated not wearing a bra with greater perceived infidelity.

Higher sociosexuality, relationship status, and sexual harassment intentions were significant positive predictors of associating not wearing a bra with infidelity. Divorced men reported the highest infidelity attributions, followed by widowed, dating, married, and single-men. Age, education level, and preference for large breasts had significant negative effects on perceived infidelity.

Removing SOI affected the model more than removing pornography consumption, indicating SOI was the stronger predictor. The model presented excludes pornography consumption.

### Comparison of preferences for (not) wearing a bra between men and women

Because the summed scores for attractiveness and fidelity ratings were not normally distributed (Shapiro–Wilk: W = 0.91 and 0.65, both *p* < 0.001), Mann–Whitney U tests were used.

**Figure 9:**
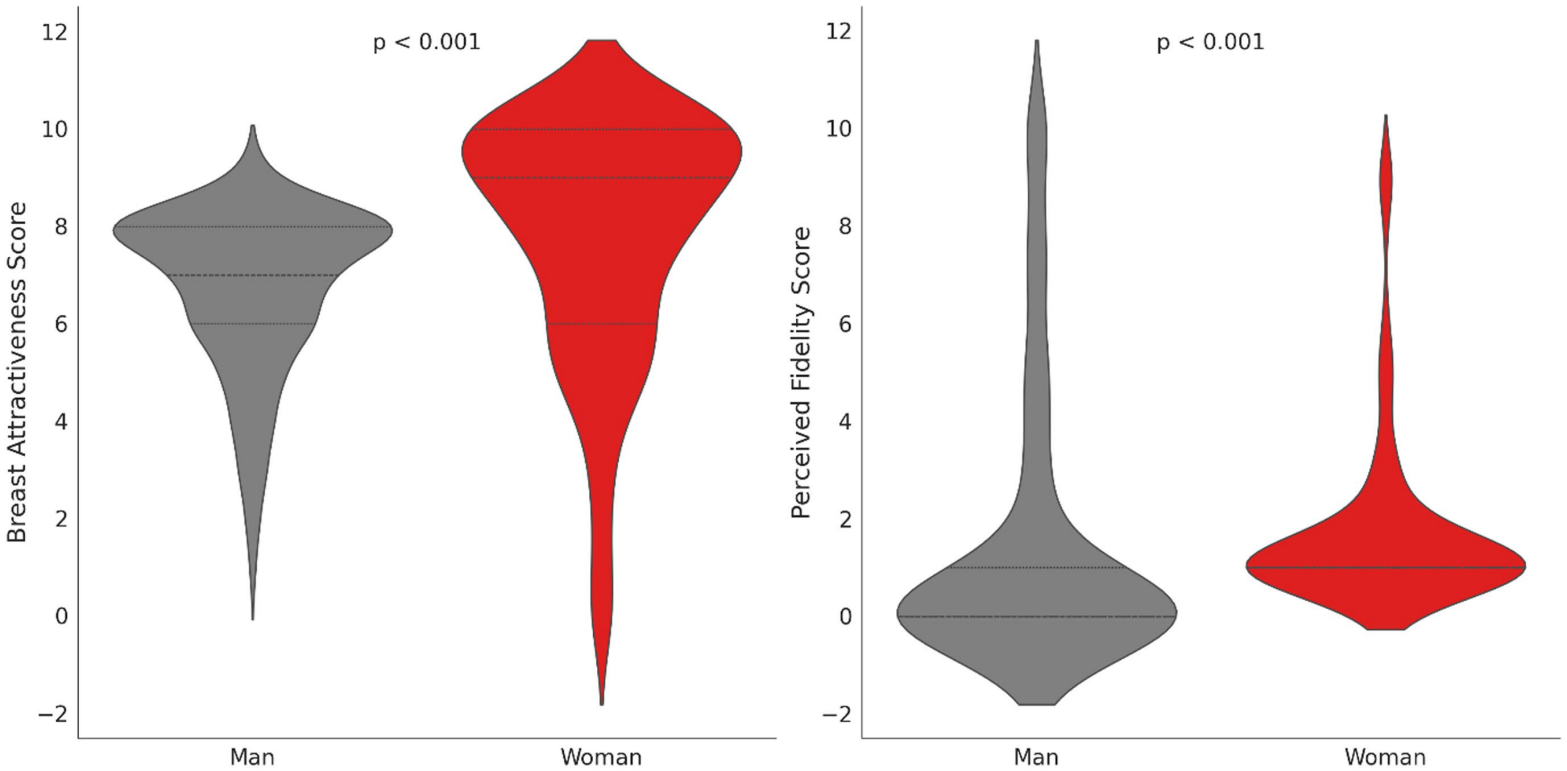
Differences between the sexes in perceived attractiveness and fidelity of breasts without a bra.

Women (*N* = 158) rated breasts without a bra as significantly more attractive (median = 9.0) than men (median = 7.0; *N* = 277; Mann–Whitney U = 13435.0). Women also associated not wearing a bra with greater fidelity (median = 1.0) than men (median = 0.0; Mann–Whitney U = 11045.0) (Figure 9).

### Additional gender differences

Men were slightly less educated than women, although the difference was negligible in practical terms (Supplementary Table 6). Men reported significantly higher self-esteem and sociosexuality, while women reported significantly greater fear of sexual harassment compared with men’s harassment intentions. Media exposure did not differ significantly between genders.

## Discussion

This study presents the first empirical investigation of the correlates of bra-wearing in women and the social perceptions of bralessness in both sexes. Our findings indicate that women are more likely to wear bras in public than in private settings, suggesting that their function extends beyond utility to include social signaling, potentially related to self-presentation. These patterns also suggest the influence of social and cultural norms: wearing a bra in public may reflect expectations about appropriateness, or professional appearance, while going braless at home likely reflects greater comfort and privacy.

The behavior of women with breast implants indirectly supports the honest signaling hypothesis. While a larger breast size is often associated with more frequent bra use (this study), our data found that a larger breast size was also correlated with higher breast dissatisfaction. Consider the positive association between having silicone implants and a greater likelihood of being braless in public settings. Implants typically increase both breast size and satisfaction with breast appearance (Chao et al., 2016), aligning with the common desire for larger breasts (reported by 47.5% of women in a cross-national survey; Swami et al., 2020). Therefore, the tendency for women with augmented—and often firmer—breasts to forgo bras contradicts the simple idea that a larger breast size leads to more coverings. Instead, it suggests that when women possess breasts they perceive as optimal (in terms of shape and firmness), they are more likely to use a braless state as a self-presentation strategy. From their perspective, their breasts function as an “honest signal” of quality and confidence, even though the signal is technically artificial. These findings also suggest that women’s decisions may be shaped by proximate psychological factors, such as self-esteem, body image, and satisfaction with breast appearance. Women with higher breast satisfaction and self-esteem were more likely to go braless, indicating that individual confidence and body comfort influence evolutionarily relevant choices about bra-wearing and breast visibility.

Our second hypothesis addressed the positive association between a braless state and sociosexual orientation (SOI). Specifically, we expected that females with greater openness to short-term mating would signal their interest in casual sex by adopting a braless strategy. However, this hypothesis was not supported. Previous research has shown that women with a more unrestricted sociosexual orientation engage more frequently in appearance-enhancement behaviors than those with a restricted orientation (Bleske-Rechek and Buss, 2006). Unrestricted sociosexuality did not correlate with the frequency of wearing high heels (Prokop and Švancárová, 2020) or the frequency of going braless (this study). This suggests that women interested in casual sex may prefer appearance-enhancement strategies other than these specific sartorial choices (Davis and Arnocky, 2022).

Alternatively, the absence of an association between sociosexuality (SOI) and bra-wearing frequency highlights the influence of non-mating-related factors. Practical considerations, fashion preferences, or habitual routines may outweigh sociosexuality in women’s daily decisions about bra use. This suggests that not all appearance-enhancing behaviors stem from mating strategies.

In line with our hypothesis, women who were braless were perceived as less faithful than those who wore a bra. This perception may be driven by the increased visibility of the nipples, which are associated with sexual arousal and perceived sexual accessibility (Burch and Widman, 2021; Widman and Burch, 2024). This aligns with broader findings that certain physical traits, such as larger breast size, are perceived as signaling greater promiscuity and lower intelligence (Kościński et al., 2020). Both phenomena share a common thread of increased visual salience: larger breasts are naturally more conspicuous, whereas the braless state deliberately enhances the visibility and shape of the breasts.

Consequently, going braless appears to function as a form of sexual signaling, analogous to the use of sexually suggestive colors or high-heeled shoes (Elliot et al., 2013; Prokop and Švancárová, 2020). Otherwise, wearing a bra can be used to conceal intimate body parts, which is a form of sexual restrictiveness and fidelity (Pazhoohi and Kingstone, 2020; Pazhoohi, 2024). This suggests a strategic trade-off between the two. While this signal may be advantageous in short-term mating contexts by emphasizing sexual availability, it simultaneously carries a potential cost for long-term relationships by reducing the perceived fidelity and commitment of the signaler.

The finding that fear of sexual harassment was negatively associated with going braless supports our hypothesis, which is grounded in the tendency to blame women for wearing provocative attire (Johnson and Workman, 1992; Wolfendale, 2016). This suggests that women not wearing a bra are perceived not only as more sexually attractive, a perception consistently supported by both male and female ratings, but also as more sexually available and, consequently, more vulnerable to sexual advances and sexual assault. Women appear to be intuitively aware of these associations, reinforcing the idea that bras can function as a form of coverage for body parts that have evolved into sexual signals. In line with evolutionary reasoning, female participants associated the braless state with higher fidelity than male participants did. This perceptual difference can be explained by heightened jealousy in response to sexual infidelity in males, which is largely attributed to concerns over paternal certainty (Edlund et al., 2019).

These findings also highlight the proximal role of social risk perception in clothing decisions. Concerns about harassment appear to limit women’s willingness to display sexualized cues in public. This reflects learned social responses, which women may have readily adopted due to evolutionary predispositions to avoid sexual threats. Media exposure and education may likewise shape norms and expectations about appropriate dress, further interacting with evolutionarily relevant cues.

Interestingly, males with harassment intentions were significantly more sensitive to the braless state as a cue to infidelity than to sexual attractiveness. This is consistent with research linking sexual harassment to jealousy (Logan and Landhuis, 2023; Nocentini et al., 2023). From an evolutionary perspective, this focus on infidelity rather than mere attractiveness is highly valid. The core problem of paternity uncertainty is hypothesized to have selected for the emotion of male sexual jealousy, which motivates behaviors aimed at mate retention (Kaighobadi et al., 2009). Jealousy deters rivals and prevents partner infidelity, thereby mitigating the risk of paternity loss (Buss et al., 1992; Daly et al., 1982). Consequently, for men predisposed to harassment, a braless state may not simply signal attraction but may be interpreted through the lens of this deep-seated jealousy, triggering perceptions of sexual exploitability and a higher likelihood of infidelity that facilitates coercive behavior.

Beyond evolutionary accounts, these gender differences may also stem from culturally transmitted beliefs about sexuality, morality, and propriety. Men can be learned to link exposed breasts to sexual availability or infidelity, whereas women view the same cues in terms of self-confidence, peer comparison, or rivalry with other women. Such proximate social and cognitive factors flesh out evolutionary explanations and explain the variability we see in perceptions.

In line with evolutionary reasoning, females associated breasts without a bra with greater fidelity than did males. This difference could again be explained by the greater pressure toward paternity certainty imposed on males than on females.

The unexpected finding that women rated braless breasts as more attractive than men did can be interpreted through the mechanism of intrasexual competition: while male ratings may reflect predominantly sexual interest, female ratings likely reflect sensitivity to cues of fertility, femininity, and social status. Women assess the mate value of potential rivals (Buss and Schmitt, 1993), and a braless state may be perceived as a signal of confidence and high mate value, leading to higher attractiveness ratings in a competitive context. This suggests that the perception of a braless state is not purely a sexual signal from women to men but also a socially relevant signal among women. This area of research requires further attention.

## Limitation

One of the limitations of our research is that the data on bra-wearing frequency and media exposure were collected via self-report. This method is subjective and may be susceptible to recall inaccuracies and social desirability bias. For instance, participants may have underreported or overreported their bra-wearing habits based on perceived social norms, or may not have accurately remembered their frequency of media consumption. However, the primary aim of our study was to examine relationships between variables, not absolute prevalence rates. Future research can employ more objective methodologies, such as a diary study, which could capture bra-wearing habits in real-time, reducing possible memory errors.

Secondly, our study frames bra-wearing in evolutionary terms, but future work should directly test cultural, normative, and psychological drivers of clothing choices. Cross-cultural studies, experiments varying social context, and assessments of fashion tastes or body image would reveal how these factors interplay with evolved sexual signals.

## Conclusion

These findings suggest that the braless state functions as a salient social signal, consistently perceived as more attractive yet also as less faithful by both sexes, suggesting a strategic trade-off for women: while going braless may enhance sexual appeal, it simultaneously incurs social costs by increasing perceptions of sexual exploitability and infidelity, which are directly linked to a heightened fear of sexual harassment. The stark contrast in the frequency of bra-wearing between public and private environments strongly suggests that women consciously manipulate the visibility of their breasts, indicating that the braless state can function as an intentional sexual signal. Women with smaller, firmer breasts are more likely to go braless, challenging the simplistic assumption that “bigger is better” and supporting the hypothesis that breast firmness, as an honest cue of youth and nulliparity, is the most important sexual signal. The heightened sensitivity of men with harassment intentions to infidelity cues, rather than mere attractiveness, in a braless woman reveals how this signal can be moderated by male sexual jealousy and ultimately, paternity uncertainty. Conversely, the higher attractiveness ratings given by women to braless peers suggest that wearing a bra is influenced by intrasexual competition among women.

## Data Availability

The original contributions presented in the study are included in the article/Supplementary material, further inquiries can be directed to the corresponding author.
