# Supplementary material for: Women’s and men’s perceptions of (not) wearing a bra in public
Source: Front Psychol. 2026 Apr 13;17:1797201. doi: 10.3389/fpsyg.2026.1797201 (PMC13111392; doi:10.3389/fpsyg.2026.1797201)
Supplement: Supplementary file 1 [file Table_1.docx]

**Supplementary Table 1.** Frequency of wearing a bra in public and at home by women

|  | **Wearing a Bra in Public (N)** | **Wearing a Bra in Public (%)** | **Wearing a Bra at Home (N)** | **Wearing a Bra at Home (%)** |
| --- | --- | --- | --- | --- |
| Never | 321 | 78.5 | 109 | 26.7 |
| 2-5x per month | 50 | 12.2 | 76 | 18.6 |
| 6-10x per month | 18 | 4.4 | 52 | 12.7 |
| 11-24x per month | 11 | 2.7 | 39 | 9.5 |
| 25x per month and more | 9 | 2.2 | 133 | 32.5 |
| Total | 409 | 100 | 409 | 100 |

**Supplementary Table 2**. Full GLMM on wearing a bra in public by women

| **Variable** | **Coefficient** | **Std_Error** | **z_value** | **p_value** | **CI_025** | **CI_975** |
| --- | --- | --- | --- | --- | --- | --- |
| Intercept | 0.892677 | 0.055485 | 16.08865 | 3.06E-58 | 0.783929 | 1.001425 |
| Age_centered | -0.0064 | 0.001717 | -3.72667 | 0.000194 | -0.00976 | -0.00303 |
| Education_Level_centered | -0.08614 | 0.013647 | -6.31171 | 2.76E-10 | -0.11288 | -0.05939 |
| Self_esteem_centered | 0.029692 | 0.009264 | 3.205 | 0.001351 | 0.011534 | 0.04785 |
| SOI_total_centered | 0.000546 | 0.00149 | 0.366451 | 0.714028 | -0.00237 | 0.003465 |
| Media_Exposure_centered | -0.01188 | 0.002749 | -4.32175 | 1.55E-05 | -0.01727 | -0.00649 |
| Sexual_Harassment_centered | -0.13025 | 0.016169 | -8.05551 | 7.91E-16 | -0.16194 | -0.09856 |
| Breast_Size_centered | -0.01559 | 0.005607 | -2.78027 | 0.005431 | -0.02658 | -0.0046 |
| Relationship_Status_centered | -0.02785 | 0.01485 | -1.8752 | 0.060765 | -0.05695 | 0.001259 |
| Pornography_Consumption_centered | 0.007197 | 0.01828 | 0.393718 | 0.69379 | -0.02863 | 0.043026 |
| Breast_Shape | 0.059771 | 0.018099 | 3.302486 | 0.000958 | 0.024298 | 0.095243 |
| Breast_Dissatisfaction_Pictures | -0.02807 | 0.005553 | -5.05467 | 4.31E-07 | -0.03895 | -0.01718 |
| Breast_Satisfaction_Items | 0.070884 | 0.009774 | 7.252556 | 4.09E-13 | 0.051728 | 0.090041 |
| Silicon_implants | 0.455014 | 0.057538 | 7.908067 | 2.61E-15 | 0.342241 | 0.567786 |
| Group Var | 1.45E+10 | 3.37E+08 | 42.89995 | 0 | 1.38E+10 | 1.51E+10 |

**Supplementary Table 3.** Correlation matrice for womens items sorted by strength of correlations (N = 409). * P <0.05, ** P < 0.01, *** P<0.001.

| **Variable 1** | **Variable 2** | **r** | **p** |
| --- | --- | --- | --- |
| Breast dissatisfaction Picture | Breast Size | -0.748*** | < .001 |
| Breast Shape | Breast Size | -0.614*** | < .001 |
| Breast dissatisfaction Picture | Breast Shape | 0.495*** | < .001 |
| Breast Satisfaction Items | Self-confidence | 0.408*** | < .001 |
| Breast Satisfaction Items | Breast Shape | 0.365*** | < .001 |
| Pornography Consumption | SOI total | 0.276*** | < .001 |
| Age | Breast Shape | -0.249*** | < .001 |
| Age | Breast Size | 0.223*** | < .001 |
| Breast Satisfaction Items | Silicon implants | 0.222*** | < .001 |
| Age | Education Level | 0.190*** | < .001 |
| Breast Size | Sexual Harassment | 0.188*** | < .001 |
| Age | Breast dissatisfaction Picture | -0.178*** | < .001 |
| Breast dissatisfaction Picture | Sexual Harassment | -0.173*** | < .001 |
| Self-confidence | Silicon implants | 0.173*** | < .001 |
| Breast Satisfaction Items | Breast dissatisfaction Picture | 0.162** | 0.001 |
| Breast Satisfaction Items | Breast Size | -0.149** | 0.003 |
| Age | Relationship Status | 0.137** | 0.005 |
| Breast Shape | Sexual Harassment | -0.134** | 0.007 |
| Age | Breast Satisfaction Items | 0.133** | 0.007 |
| Breast Shape | SOI total | -0.133** | 0.007 |
| Breast Shape | Self-confidence | 0.131** | 0.008 |
| Age | Self-confidence | 0.129** | 0.009 |
| Breast Size | Self-confidence | -0.122* | 0.014 |
| Breast dissatisfaction Picture | Self-confidence | 0.119* | 0.016 |
| SOI total | Self-confidence | 0.115* | 0.020 |
| Education Level | Media Exposure | 0.102* | 0.038 |
| Education Level | Sexual Harassment | -0.096 | 0.051 |
| Relationship Status | SOI total | -0.090 | 0.068 |
| Education Level | SOI total | 0.087 | 0.080 |
| SOI total | Silicon implants | 0.085 | 0.085 |
| Age | Media Exposure | 0.083 | 0.093 |
| Breast Size | Silicon implants | 0.079 | 0.109 |
| Breast Shape | Pornography Consumption | -0.073 | 0.143 |
| Breast Shape | Relationship Status | -0.072 | 0.146 |
| Self-confidence | Sexual Harassment | -0.070 | 0.158 |
| Breast Satisfaction Items | Education Level | 0.068 | 0.170 |
| Breast Size | SOI total | 0.067 | 0.179 |
| Pornography Consumption | Sexual Harassment | -0.066 | 0.182 |
| Breast Shape | Education Level | 0.065 | 0.187 |
| Age | Pornography Consumption | -0.064 | 0.197 |
| SOI total | Sexual Harassment | -0.062 | 0.214 |
| Age | Silicon implants | 0.056 | 0.256 |
| Pornography Consumption | Relationship Status | -0.055 | 0.268 |
| Age | SOI total | 0.053 | 0.283 |
| Breast Shape | Silicon implants | 0.052 | 0.290 |
| Breast Satisfaction Items | Sexual Harassment | -0.052 | 0.292 |
| Relationship Status | Sexual Harassment | 0.051 | 0.301 |
| Breast Satisfaction Items | SOI total | -0.048 | 0.334 |
| Education Level | Relationship Status | 0.047 | 0.346 |
| Breast Size | Media Exposure | 0.046 | 0.348 |
| Breast Size | Pornography Consumption | 0.045 | 0.369 |
| Education Level | Silicon implants | -0.043 | 0.381 |
| Sexual Harassment | Silicon implants | -0.042 | 0.399 |
| Breast Size | Relationship Status | 0.041 | 0.412 |
| Breast dissatisfaction Picture | Media Exposure | -0.040 | 0.415 |
| Media Exposure | Sexual Harassment | -0.040 | 0.422 |
| Pornography Consumption | Self-confidence | 0.040 | 0.424 |
| Relationship Status | Self-confidence | 0.039 | 0.437 |
| Age | Sexual Harassment | 0.035 | 0.475 |
| Media Exposure | Silicon implants | -0.033 | 0.509 |
| Breast Shape | Media Exposure | -0.031 | 0.538 |
| Education Level | Pornography Consumption | -0.029 | 0.561 |
| Media Exposure | Relationship Status | 0.027 | 0.583 |
| Media Exposure | Self-confidence | -0.025 | 0.610 |
| Breast dissatisfaction Picture | Relationship Status | -0.025 | 0.620 |
| Education Level | Self-confidence | 0.022 | 0.656 |
| Breast dissatisfaction Picture | Pornography Consumption | -0.018 | 0.723 |
| Breast dissatisfaction Picture | Silicon implants | 0.016 | 0.753 |
| Breast Satisfaction Items | Relationship Status | -0.015 | 0.759 |
| Breast Satisfaction Items | Media Exposure | 0.011 | 0.819 |
| Breast Size | Education Level | -0.011 | 0.827 |
| Breast dissatisfaction Picture | SOI total | -0.011 | 0.832 |
| Relationship Status | Silicon implants | -0.010 | 0.834 |
| Media Exposure | Pornography Consumption | -0.007 | 0.880 |
| Breast dissatisfaction Picture | Education Level | -0.007 | 0.894 |
| Pornography Consumption | Silicon implants | 0.005 | 0.914 |
| Breast Satisfaction Items | Pornography Consumption | -0.005 | 0.920 |
| Media Exposure | SOI total | -0.005 | 0.924 |

**Supplementary Table 4.** Full GLMM on wearing a bra at home by women

| **Variable** | **Coefficient** | **Std_Error** | **z_value** | **p_value** | **CI_025** | **CI_975** |
| --- | --- | --- | --- | --- | --- | --- |
| Intercept | 2.314258 | 0.103405 | 22.38059 | 6.1E-111 | 2.111589 | 2.516928 |
| Age_centered | -0.01814 | 0.003199 | -5.66885 | 1.44E-08 | -0.02441 | -0.01187 |
| Education_Level_centered | 0.149058 | 0.025434 | 5.860683 | 4.61E-09 | 0.099209 | 0.198907 |
| Self_esteem_centered | 0.0088 | 0.017266 | 0.509709 | 0.610255 | -0.02504 | 0.04264 |
| SOI_total_centered | 0.009417 | 0.002776 | 3.392348 | 0.000693 | 0.003976 | 0.014858 |
| Media_Exposure_centered | -0.01455 | 0.005123 | -2.84099 | 0.004497 | -0.0246 | -0.00451 |
| Sexual_Harassment_centered | -0.12074 | 0.030133 | -4.00688 | 6.15E-05 | -0.1798 | -0.06168 |
| Breast_Size_centered | -0.08253 | 0.010449 | -7.89811 | 2.83E-15 | -0.10301 | -0.06205 |
| Relationship_Status_centered | -0.02195 | 0.027676 | -0.79303 | 0.427758 | -0.07619 | 0.032296 |
| Pornography_Consumption_centered | 0.002192 | 0.034068 | 0.064331 | 0.948707 | -0.06458 | 0.068964 |
| Breast_Shape | -0.05015 | 0.03373 | -1.48688 | 0.137046 | -0.11626 | 0.015957 |
| Breast_dissatisfaction_Picture | -0.02244 | 0.010349 | -2.16794 | 0.030164 | -0.04272 | -0.00215 |
| Breast_Satisfaction_Items | 0.186694 | 0.018215 | 10.24951 | 1.19E-24 | 0.150994 | 0.222395 |
| Silicon_implants | 0.957994 | 0.10723 | 8.934031 | 4.11E-19 | 0.747827 | 1.16816 |
| Group Var | 1.45E+10 | 3.37E+08 | 42.9009 | 0 | 1.38E+10 | 1.51E+10 |

**Supplementary Table 5.** Full GLMM on mens preference for attractiveness of women pictures not wearing a bra

| **Variable** | **Standardized Coefficient** | **Std_Error** | **p_value** | **Significance** | **Odds Ratio** | **OR_CI_Lower** | **OR_CI_Upper** |
| --- | --- | --- | --- | --- | --- | --- | --- |
| Breast Size Preference_centered | 0.143455 | 0.050876 | 0.004678 | ** | 1.154255 | 1.044708 | 1.275288 |
| Age_centered | 0.082806 | 0.05706 | 0.145184 |  | 1.086331 | 0.971385 | 1.214879 |
| Education level_centered | -0.06177 | 0.053251 | 0.244054 |  | 0.940103 | 0.84693 | 1.043526 |
| Relationship Status_centered | 0.048511 | 0.053201 | 0.360649 |  | 1.049707 | 0.945762 | 1.165075 |
| Sex Harassment_centered | 0.031985 | 0.052703 | 0.543618 |  | 1.032502 | 0.93117 | 1.144862 |
| Media Exposure_centered | -0.01577 | 0.053793 | 0.767935 |  | 0.98435 | 0.885849 | 1.093803 |
| Porn consumption | 0.011762 | 0.051256 | 0.817295 |  | 1.011831 | 0.915119 | 1.118763 |
| SOI total_centered | 0.007677 | 0.053415 | 0.886531 |  | 1.007707 | 0.907542 | 1.118926 |
| Self-esteem_centered | -0.00479 | 0.050338 | 0.923876 |  | 0.995226 | 0.901723 | 1.098426 |

**Supplementary Table 6.** Full GLMM on mens preference for fidelity of women pictures not wearing a bra

| **Feature** | **Coefficient** | **Std_Error** | **p_value** | **Significance** | **Odds Ratio** | **OR_CI_Lower** | **OR_CI_Upper** |
| --- | --- | --- | --- | --- | --- | --- | --- |
| Age_centered | -0.05513 | 0.079753 | 8.38E-09 | *** | 0.946362 | 0.809414 | 1.106482 |
| Education level_centered | -0.23698 | 0.073853 | 0.000757 | *** | 0.789004 | 0.682675 | 0.911894 |
| Self-esteem_centered | 0.053087 | 0.067532 | 0.286631 |  | 1.054521 | 0.923785 | 1.203759 |
| SOI total_centered | 0.02495 | 0.070521 | 1.48E-05 | *** | 1.025264 | 0.892909 | 1.177237 |
| Media Exposure_centered | -0.01352 | 0.071766 | 0.327668 |  | 0.986571 | 0.857117 | 1.135578 |
| Sex Harassment_centered | 0.536004 | 0.059896 | 1.27E-07 | *** | 1.709163 | 1.519844 | 1.922064 |
| Breast Size Preference_centered | -0.07038 | 0.070608 | 0.002717 | ** | 0.932043 | 0.811584 | 1.070382 |
| Relationship Status_centered | 0.350645 | 0.070279 | 1.99E-06 | *** | 1.419984 | 1.237258 | 1.629695 |

**Supplementary Table 7.** Additional gender differences

|  |
| --- |

| **Variable** | **Men (N)** | **Men Mean (SD)** | **Men Median** | **Women (N)** | **Women Mean (SD)** | **Women Median** | **Test Used** | **p-value** | **Significance** |
| --- | --- | --- | --- | --- | --- | --- | --- | --- | --- |
| Age | 277 | 35.581 (8.333) | 35.0 | 409 | 34.002 (8.32) | 33.0 | Mann-Whitey U | 0.009 | ** |
| Education leel | 277 | 3.043 (1.038) | 3.0 | 409 | 3.296 (0.984) | 4.0 | Mann-Whitey U | 0.001 | ** |
| Self-esteem | 277 | 4.394 (1.351) | 4.0 | 409 | 3.753 (1.562) | 4.0 | Mann-Whitey U | 0.0 | *** |
| SOI total | 277 | 36.881 (12.209) | 37.0 | 409 | 26.572 (9.307) | 26.0 | Mann-Whitey U | 0.0 | *** |
| Media exposure | 277 | 18.84 (5.14) | 18.0 | 409 | 18.04 (4.73) | 18.0 | Mann-Whitey U | 0.0 | *** |
| Sex Harassment | 277 | 1.543 (0.583) | 1.357 | 409 | 2.259 (0.821) | 2.214 | Mann-Whitey U | 0.0 | *** |

**Supplementary Table Z.** Correlation matrice for men items sorted by strength of correlations (N = 277). * P <0.05, ** P < 0.01, *** P<0.001.

| **Variable 1** | **Variable 2** | **Correlation** | **Significance** | **p_value** |
| --- | --- | --- | --- | --- |
| Age_centered | Relationship Status_centered | 0.3786 | *** | 0.0000 |
| Age_centered | Media Exposure_centered | 0.2994 | *** | 0.0000 |
| Age_centered | Education level_centered | 0.2923 | *** | 0.0000 |
| SOI total_centered | Sex Harassment_centered | 0.2273 | *** | 0.0001 |
| Education level_centered | Media Exposure_centered | 0.2269 | *** | 0.0001 |
| Education level_centered | Relationship Status_centered | 0.2072 | *** | 0.0005 |
| Sex Harassment_centered | Breast Size Preference_centered | 0.1964 | ** | 0.0010 |
| SOI total_centered | Breast Size Preference_centered | 0.1933 | ** | 0.0012 |
| SOI total_centered | Media Exposure_centered | 0.1871 | ** | 0.0018 |
| Self-esteem_centered | SOI total_centered | 0.1750 | ** | 0.0035 |
| Education level_centered | Sex Harassment_centered | -0.1630 | ** | 0.0066 |
| Media Exposure_centered | Relationship Status_centered | 0.1402 | * | 0.0196 |
| Self- esteem _centered | Media Exposure_centered | 0.1178 |  | 0.0502 |
| Self- esteem _centered | Breast Size Preference_centered | 0.1158 |  | 0.0541 |
| Media Exposure_centered | Sex Harassment_centered | 0.1067 |  | 0.0762 |
| Self- esteem _centered | Relationship Status_centered | 0.0942 |  | 0.1176 |
| Sex Harassment_centered | Relationship Status_centered | -0.0795 |  | 0.1870 |
| Self- esteem _centered | Sex Harassment_centered | 0.0697 |  | 0.2475 |
| Education level_centered | Self-esteem_centered | 0.0679 |  | 0.2603 |
| Age_centered | SOI total_centered | 0.0667 |  | 0.2685 |
| Media Exposure_centered | Breast Size Preference_centered | 0.0546 |  | 0.3656 |
| Education level_centered | SOI total_centered | 0.0536 |  | 0.3744 |
| Breast Size Preference_centered | Relationship Status_centered | -0.0475 |  | 0.4315 |
| Age_centered | Breast Size Preference_centered | 0.0335 |  | 0.5784 |
| Age_centered | Self-esteem_centered | 0.0221 |  | 0.7144 |
| Education level_centered | Breast Size Preference_centered | -0.0216 |  | 0.7210 |
| SOI total_centered | Relationship Status_centered | 0.0113 |  | 0.8516 |
| Age_centered | Sex Harassment_centered | -0.0055 |  | 0.9275 |
